# GAMPoint: Geometry-augmented vision transformer with a multimodal-ready interface for 3D point-cloud segmentation

**DOI:** 10.1371/journal.pone.0360907

**Published:** 2026-10-23

**Authors:** Xinyu Mu, Xuan He, Ziqi Huang, Ting Liu

**Affiliations:** 1 Department of Artificial Intelligence, School of Artificial Intelligence, Guangzhou Institute of Science and Technology, Guangzhou, China; 2 South China Normal University Affiliated Huadu School, Guangzhou, China; 3 Faculty of Science and Technology, University of Macau, Macau, China; Centro de Investigacion en Ciencias de Informacion Geoespacial AC (Research Center on Geospatial Information Sciences), MEXICO

## Abstract

Various 3D point cloud semantic segmentation methods have been proposed, yet they remain challenged by limited use of geometric cues and inaccurate predictions around thin structures, object boundaries, and rare classes in cluttered indoor scenes. To address these limitations, we propose GAMPoint, a transformer-based framework for 3D semantic segmentation. Specifically, Enhanced Relative Positional Encoding (EnhancedRPE) augments relative positional modeling with local surface-variation cues to strengthen geometry-aware feature learning. In addition, Conditional Multimodal Gating (CMG) is introduced as an input-side embedding interface. In GAMPoint, XYZ coordinates provide the basic point-cloud geometry, while CMG incorporates available auxiliary attributes, such as RGB colors and surface normals, before the serialized encoder. For RGB-D reconstructed datasets such as ScanNet, XYZ coordinates already contain depth-derived geometric information; raw depth is not used as an additional per-point channel. GAMPoint incorporates EnhancedRPE and CMG into Point Transformer V3 and is evaluated on S3DIS and ScanNet. Experimental results show competitive performance and modest gains over strong point-transformer baselines. Class-wise and repeated-run analyses further indicate category-dependent improvements and consistent gains across matched repeated runs under the reported settings. Code is available at github.com/airmanss/ GAMPoint.

## Introduction

3D point cloud semantic segmentation is a fundamental task for 3D scene understanding, with common application to autonomous driving, robotics perception, immersive scene capture, digital-twin modeling, and industrial inspection [1–4]. Traditional methods were proposed for point cloud segmentation by typically relying on handcrafted geometric cues and local and global descriptors, such as binary or rotation-invariant 3D features and supervoxel-based partitioning [5–7]. However, they were tailored to specific sensor characteristics and scene statistics. To address these limitations, deep learning (DL) methods were developed as data-driven representations, and these DL-based point-wise networks and voxel/sparse-convolutional backbones substantially improved accuracy and scalability on large scenes [8–10]. Additionally, vision transformer (ViT)-based methods utilize attention mechanisms to aggregate contextual information globally [11]. However, modeling long-range interactions in irregular and non-uniform point sets remains challenging. Specifically, the Point Transformer and its variants were proposed as efficient and strong ViT-based baselines for indoor semantic segmentation [12–14], but they still have limited mechanisms for explicitly using geometric features and may produce inaccurate predictions around thin structures, boundaries, and rare classes under occlusion and cluttered indoor scenes.

Existing DL point cloud segmentation methods mainly differ in the way of representing and processing raw point sets. First, indirect methods map point clouds to structured intermediates (multi-view images or voxel grids), thus enabling the models to utilize convolutional operators to extract features for segmentation [9,15]. However, their performance depends on the selection and alignment of views, and thus are limited by discretization artifacts and large empty space. Second, point-based models preserve continuous geometric information by avoiding heavy pre-processing. Thus, they are required to handle irregular structures, non-uniform sampling, and sensor noise. However, these factors make it difficult for these models to encode higher-order surface variation with high robustness, especially near boundaries and in thin objects [8,16,17]. In addition to point coordinates, indoor point-cloud data often provide auxiliary attributes such as RGB colors and surface normals, which offer complementary appearance and surface-orientation cues [18–20]. These observations motivate an input-side embedding that can combine coordinates with available auxiliary attributes in a unified form.

To address these limitations, we propose a geometry-augmented vision transformer with a multimodal-ready interface for 3D point cloud segmentation, termed **GAMPoint**. Specifically, we propose an **Enhanced Relative Positional Encoding** (EnhancedRPE) module to augment relative positional modeling with local surface variation cues, thus enabling the segmentation models to utilize more geometric information. It combines normal-angle and curvature differences into serialized self-attention to strengthen geometry-aware modeling on boundaries, thin structures, and planar/curved regions. Additionally, we propose a **Conditional Multimodal Gating** (CMG) module as an input-side embedding interface that combines point coordinates with available auxiliary attributes, such as RGB colors and surface normals, before the serialized encoder. We constructed the GAMPoint by incorporating the EnhancedRPE and CMG modules into the Point Transformer V3 backbone. To evaluate the effectiveness of the GAMPoint, we evaluated it on two commonly used indoor point cloud segmentation benchmarks, including S3DIS and ScanNet. The experimental results show that GAMPoint achieves competitive performance and provides modest gains over strong point-transformer baselines. Fig 1 demonstrates an overview of the GAMPoint framework and a high-level comparison with SOTA methods. Additionally, class-wise and repeated-run analyses are used to characterize the category dependence and run-to-run variability of the observed performance gains.

**Fig 1:**
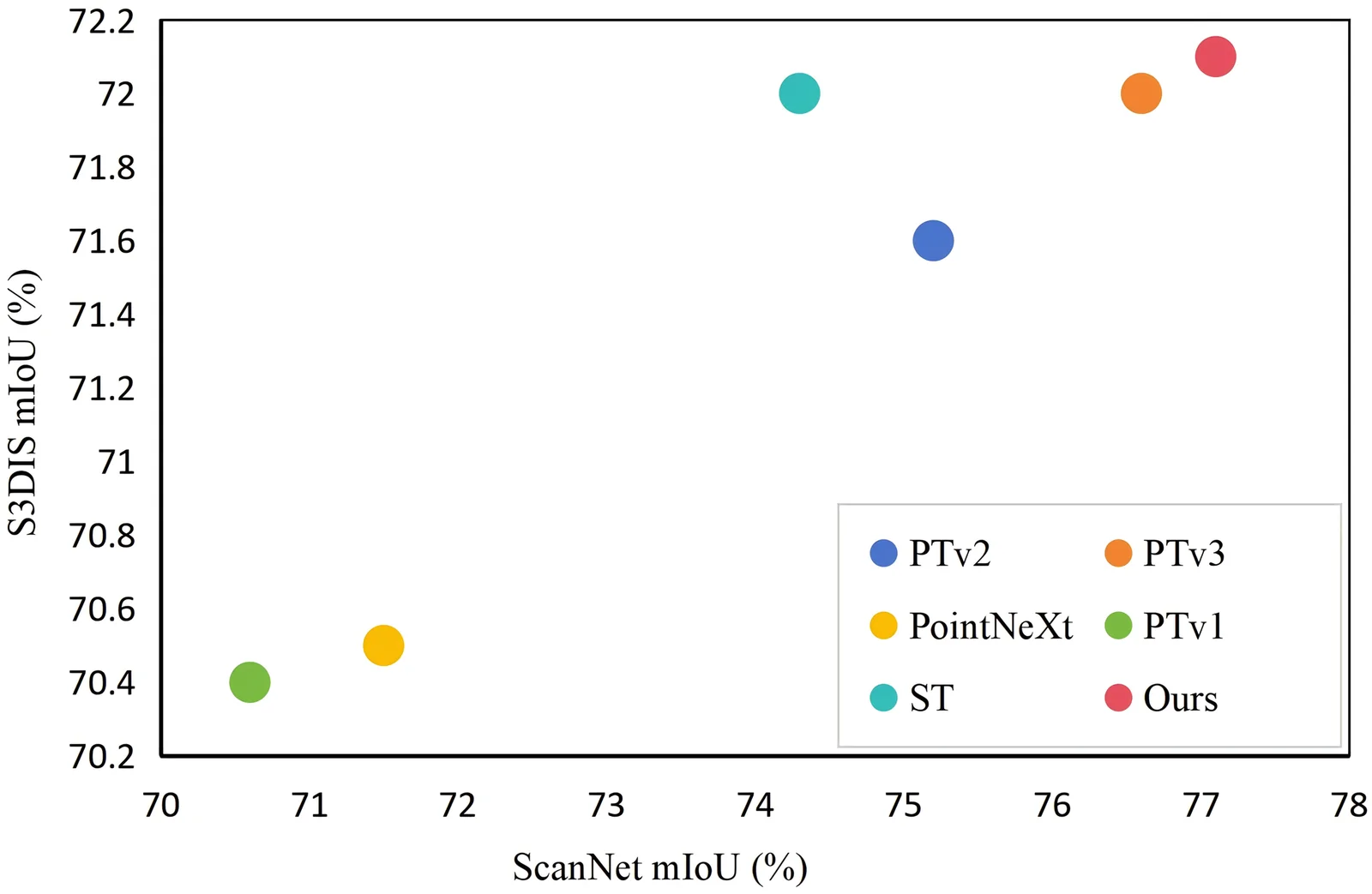
Performance comparison of GAMPoint (Ours) and representative baselines on ScanNet and S3DIS. Each point denotes one method, plotted by ScanNet mIoU (%) on the x-axis and S3DIS mIoU (%) on the y-axis. The PointNet and Stratified Transformer points are included as external reference baselines using the values reported in their original publications [8,21].

The main contributions are summarized as follows:

- We propose EnhancedRPE to strengthen geometry-aware relation modeling in serialized attention by incorporating local surface-variation cues.
- We propose CMG as an input-side interface for integrating XYZ, RGB, and normal attributes without using raw depth as an extra input channel.
- We develop GAMPoint by incorporating EnhancedRPE and CMG into the Point Transformer V3 backbone for 3D point-cloud segmentation.
- Experimental results on S3DIS and ScanNet show that GAMPoint achieves competitive performance and provides modest gains over strong point-transformer baselines, with category-dependent improvements in several geometry-sensitive classes.

## Related work

### Indirect representation learning

Indirect representation learning maps irregular point sets to structured intermediates, such as multi-view images, voxel grids, or sparse voxel representations, so that mature convolutional operators can be reused. Multi-view methods first project 3D data onto several image planes and then aggregate view-wise features. MVCNN [15] is an early representative method that performs view pooling over multiple 2D projections for 3D shape recognition. 3DMV [22] further combines RGB images and 3D geometry for semantic scene segmentation, showing the value of joint 2D–3D representation learning. Voint Cloud [23] reformulates point clouds with multi-view representations and explicitly considers view-wise feature aggregation. Self-supervised multi-view rendering has also been used as a pretext task for point-cloud analysis [24], while MM-3DScene [25] introduces masked modeling and self-distilled consistency for 3D scene understanding. These methods demonstrate that projected or rendered views can provide useful appearance and contextual cues. However, the projection process may also lose part of the native geometric neighborhood structure of the original point cloud.

Multimodal and cross-representation methods further improve indirect representation learning by combining point-cloud features with image or view-based cues. Hybrid3D [18] jointly learns point-cloud and multi-view image features for registration. MFFNet [26] designs a multimodal feature fusion network that combines point-cloud and image features for semantic segmentation. PointDC [27] uses cross-modal distillation and super-voxel clustering for unsupervised 3D semantic segmentation. Point-attention Net [19] introduces graph attention convolution to enhance point-cloud segmentation. These methods indicate that auxiliary views or image-level information can complement point-wise geometry, especially when appearance or contextual information is important.

Voxel-based methods discretize 3D space into regular or sparse grids and then apply 3D convolutions. Early dense-voxel models, such as VoxNet [9] and 3D ShapeNets [28], established the volumetric representation paradigm for 3D recognition. However, dense voxelization is computationally expensive because empty space is also represented. To reduce this cost, several more efficient structures have been proposed. Kd-Networks [29] organize points with k-d trees, and octree-guided CNNs [30] use hierarchical octree grids to reduce redundant computation. Submanifold Sparse ConvNet [10] computes convolutions only on active voxels, while focal sparse convolution [31] dynamically emphasizes important sparse locations. Minuet [32] further improves the efficiency of sparse 3D convolutions on GPUs. PCSCNet [33] combines point convolution and sparse convolution for fast LiDAR point-cloud segmentation, providing another example of hybrid point–voxel representation learning.

Overall, indirect representation learning improves computational regularity and enables the reuse of mature 2D or 3D convolutional operators. Nevertheless, these pipelines usually depend on projection, rendering, voxelization, or sparse discretization. Such transformations can weaken fine local geometry and boundary details. In contrast, our work keeps a point-transformer backbone and focuses on strengthening geometry-aware modeling within the native point-cloud representation.

### Native point cloud processing

Native point cloud processing operates directly on raw point sets, which helps reduce preprocessing complexity and avoid the information loss caused by projection or voxelization. A representative line of work learns point-wise features with shared MLPs and aggregation functions. PointNet [8] is a pioneering method that applies shared MLPs to unordered points and uses a symmetric pooling function to obtain permutation-invariant global features. PointNet++ [16] further introduces hierarchical grouping and local feature learning, which improves the ability to capture local geometric structures. However, these MLP-based methods still have limited ability to explicitly model complex neighborhood relations and high-order surface variation.

Several subsequent MLP-based variants improve point-wise representation learning by strengthening channel interaction and multi-scale feature aggregation. PointNAC [34] dynamically modulates point-wise features through copula-based channel dependency modeling. GateNet-based point-cloud networks [35] introduce gating mechanisms to control feature flow and combine multi-scale information for classification and segmentation. These methods retain the simplicity of point-wise backbones, but their geometric modeling is still mainly implicit.

Graph-based methods provide another important direction for native point-cloud processing. DGCNN [36] constructs dynamic edge features in local neighborhoods and updates graph relations during feature learning. Non-local operations [37] further inspire long-range relation modeling beyond local neighborhoods. Recent graph-based variants refine this idea in different ways. DRGCNN [38] introduces dynamic region partitioning to construct adaptive local graphs. The dual-graph attention network [39] combines attention with two graph branches, while Point-attention Net [19] uses graph attention convolution to enhance point-cloud segmentation. EDGCNet [40] combines hyperbolic graph convolution with a dual squeeze-and-attention mechanism. These graph-based designs improve local relation modeling, but repeated neighborhood construction can increase computational cost on large-scale scenes.

Convolutional operators have also been adapted to unordered point sets. PointCNN [41] learns an X-transformation to reorder local points before convolution. PointConv [42] generalizes convolution to continuous 3D space by learning density-aware convolution weights. KPConv [17] defines flexible kernel points for convolution on point clouds. CGA-Net [20] further adapts CNN-style aggregation to multimodal point-cloud features. Recent studies also explore simplified structures, lightweight pre-training, rendering-based representation learning, and point–voxel hybrid designs to improve scalability [43–45]. Overall, native point-cloud methods preserve fine geometric details better than indirect representations, but robust modeling of surface orientation, boundary variation, and long-range context remains challenging. This motivates the use of attention-based geometric modeling in our framework.

### Attention-driven optimization

Attention mechanisms and Transformer-style architectures have become important tools for improving point-cloud representation learning beyond purely MLP-, graph-, or convolution-based designs. One line of work inserts attention modules into existing point- or convolution-based backbones to refine feature aggregation. For example, local contextual attention has been used to enhance Kernel Point Convolution for 3D point-cloud semantic segmentation [46]. Other recent works improve large-scale point-cloud segmentation by combining efficient sampling or grid strategies with stronger feature aggregation modules [47]. These designs show that attention can improve semantic consistency without completely changing the underlying backbone.

A second line of work builds Transformer-style architectures for point-cloud understanding. Point Transformer [12] introduces self-attention directly into point-set processing and learns local vector attention for 3D recognition and segmentation. Point Transformer V2 [13] improves grouped vector attention and partition-based pooling. Point Transformer V3 [14] further simplifies the architecture and improves efficiency through serialized point processing. Other Transformer-based backbones also extend attention to different 3D representations. Stratified Transformer [21] uses stratified window attention for 3D point-cloud segmentation. Swin3D [48] develops a hierarchical pretrained Transformer backbone for indoor 3D scene understanding. OneFormer3D [49] explores a unified Transformer framework for different 3D segmentation tasks. These methods demonstrate the effectiveness of attention for long-range dependency modeling in 3D scenes.

A third line of work introduces explicit geometric or multimodal cues into attention-based pipelines. Transformer-based designs also target geometric feature extraction from vector shapes [50], semantic-guided multi-feature aggregation for LiDAR-based detection [51], and hybrid neural-geometric ground segmentation [52]. These studies indicate that attention can be combined with geometric priors to strengthen 3D perception.

Attention has also been widely used for multimodal fusion. Cross-modal attention-driven fusion [53] combines point clouds with other sensor information for semantic segmentation. Dual-attention fusion [54] and attention-based LiDAR–camera fusion [55] further demonstrate the usefulness of attention in 3D detection. However, many existing attention-based methods still encode local geometry mainly through raw coordinates or simple relative offsets. In contrast, our work extends the PTv3 backbone by introducing EnhancedRPE to strengthen geometry-aware attention with local surface-variation cues, and uses CMG as an input-side interface for combining point coordinates with available auxiliary point attributes.

## Method

### Overall architecture

GAMPoint is developed by incorporating EnhancedRPE and CMG into the PTv3-BaseOpt backbone, which is adopted from the official Pointcept repository [56]. The network follows a five-stage U-shaped encoder–decoder architecture for hierarchical feature learning. In this study, BaseOpt denotes the PTv3-BaseOpt baseline under the same preprocessing, input attributes, training schedule, and evaluation protocol, whereas GAMPoint denotes the corresponding model after incorporating the proposed EnhancedRPE and CMG-based input embedding. For clarity, EnhancedRPE Block (ER Block) denotes the transformer block equipped with EnhancedRPE-based serialized attention. Encoder-1 denotes the first high-resolution encoder stage after the CMG-based input embedding, whereas Encoder-5 denotes the deepest low-resolution encoder stage. Decoder-1 to Decoder-4 denote the four upsampling stages from low to high resolution. SerializedPooling and SerializedUnpooling refer to the serialized downsampling and upsampling operations inherited from the PTv3 hierarchy, respectively. Let *N* denote the number of point tokens after input embedding; the feature-map sizes (*N*/2), (*N*/4), (*N*/8), and (*N*/16) are produced by successive SerializedPooling operations. The stage-wise architecture of GAMPoint is summarized in Table 1.

**Table 1:** Stage-wise architecture summary of GAMPoint. EnhancedRPE Block (ER Block) denotes the transformer block equipped with EnhancedRPE-based serialized attention. Encoder-1 to Encoder-5 indicate the five encoder stages from high to low resolution, and Decoder-1 to Decoder-4 indicate the four decoder stages from low to high resolution. *N* denotes the number of point tokens after input embedding.

Stage	Feature-map size	Channels	ER Blocks	Attention heads	Operation
Embedding	(*N*)	32	–	–	CMG-based input embedding
Encoder-1	(*N*)	32	2	2	ER Blocks
Encoder-2	(*N*/2)	64	2	4	SerializedPooling + ER Blocks
Encoder-3	(*N*/4)	128	2	8	SerializedPooling + ER Blocks
Encoder-4	(*N*/8)	256	6	16	SerializedPooling + ER Blocks
Encoder-5	(*N*/16)	512	2	32	SerializedPooling + ER Blocks
Decoder-1	(*N*/8)	256	2	16	SerializedUnpooling + ER Blocks
Decoder-2	(*N*/4)	128	2	8	SerializedUnpooling + ER Blocks
Decoder-3	(*N*/2)	64	2	4	SerializedUnpooling + ER Blocks
Decoder-4	(*N*)	64	2	4	SerializedUnpooling + ER Blocks
Output	(*N*)	–	–	–	Linear prediction layer

**Encoder.** The encoder contains five stages, as shown in Fig 2(a). The CMG-based input embedding is used before Encoder-1, as shown in Fig 2(b), where the available point attributes are projected to 32-dimensional initial features. The five encoder stages contain 2, 2, 2, 6, and 2 ER Blocks, respectively. Their attention-head numbers are 2, 4, 8, 16, and 32, and the serialized attention-window size is fixed to *w* = 1024 across stages. SerializedPooling is applied between adjacent encoder stages to reduce the number of points and increase the feature-channel dimension.

**Fig 2:**
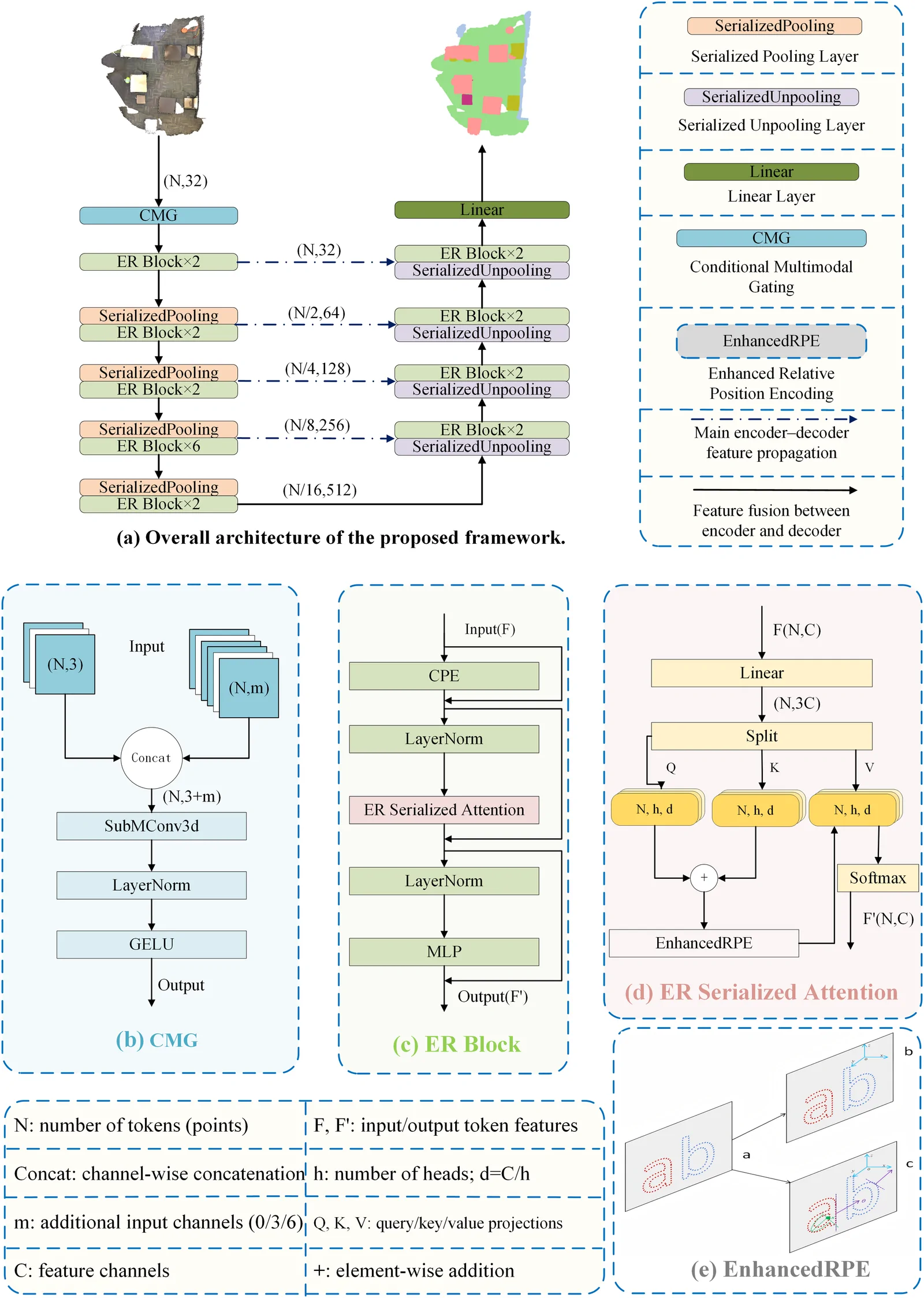
Overview of GAMPoint and the proposed modules. (a) Overall architecture of GAMPoint, a U-Net-style encoder–decoder network. The encoder and decoder consist of multiple stages with SerializedPooling/SerializedUnpooling for downsampling/upsampling, respectively, and skip connections for feature fusion. In the encoder, the numbers of ER Blocks are 2, 2, 2, 6, and 2 from shallow to deep; in the decoder, each stage uses two consecutive ER Blocks. Linear denotes a linear projection in the feature space. (b) CMG-based input embedding. XYZ coordinates provide spatial indices for sparse convolution, while RGB, surface normals, and optional aligned features are concatenated and recalibrated by learned channel-wise gating before the standard PTv3 input embedding. (c) ER Block consists of CPE, ER Serialized Attention, and an MLP module with LayerNorm and residual connections. (d) ER Serialized Attention projects features to *Q*, *K*, and *V*, splits them into *h* heads with head dimension *d* = *C*/*h*, and injects geometry-aware bias via EnhancedRPE. (e) Illustration of EnhancedRPE: (1) serialized point sequence; (2) conventional RPE using only 3D offsets; (3) EnhancedRPE incorporating both 3D offsets and surface variation cues (normal-angle and curvature).

**Decoder.** The decoder contains four stages, as shown in Fig 2(a). Each stage first upsamples the point features by SerializedUnpooling and then refines them with two ER Blocks. The decoder uses attention-head settings of 4, 4, 8, and 16, and the serialized attention-window size is also fixed to *w* = 1024. Skip connections are used to fuse the decoder features with the corresponding encoder features. Finally, a Linear layer is employed to generate point-wise predictions.

### ER Block

The ER Block follows the general architecture of a transformer block and equips the serialized attention component with EnhancedRPE-based geometric bias. As illustrated in Fig 2(c), the ER Block first applies a Convolutional Positional Encoding (CPE) layer to token features $𝐅\in {\mathbb{R}}^{N\times C}$ for position encoding in the sparse voxel space. The resulting positional features are added to the input features to form geometry-aware token embeddings. Subsequently, the ER Block stacks two functional submodules following the standard transformer pattern: an ER Serialized Attention module and a multi-layer perceptron (MLP) module. Layer normalization is applied before each of these two submodules to stabilize training. Finally, residual connections are employed around the CPE, the ER Serialized Attention module, and the MLP module, respectively, so that the ER Block preserves the input dimensionality (*N*,*C*) while enhancing geometric modeling capacity.

### ER serialized attention

ER Serialized Attention refers to the serialized self-attention operation equipped with the proposed EnhancedRPE bias, as illustrated in Fig 2(d). Specifically, given input token features $𝐅\in {\mathbb{R}}^{N\times C}$, a linear layer is employed for feature projection and generates the query, key, and value features **Q**, **K**, and **V** with the dimension of (*N*,*C*). These features are then reshaped into *h* heads, each with head dimension *d* = *C*/*h*. For each serialized token, the attention logits are computed within the fixed window as


$$\begin{array}{c} 𝐀=\frac{𝐐{𝐊}^{⊤}}{\sqrt{d}}. \end{array}$$ (1)

EnhancedRPE is employed to calculate the pair-wise geometry-aware bias *R*_ER,*ij*_, which is added to the attention logits:


$$\begin{array}{c} {\tilde{A}}_{ij}=A_{ij}+R_{ER,ij}. \end{array}$$ (2)

Finally, the output token features are computed as


$$\begin{array}{c} {𝐅}^{\prime }=\mathrm{softmax}(\tilde{𝐀})\;𝐕\in {\mathbb{R}}^{N\times C}. \end{array}$$ (3)

### Enhanced relative positional encoding module

EnhancedRPE augments the attention mechanism with explicit geometric cues, as illustrated in Fig 2(e). For a neighboring pair of points (*i*,*j*), EnhancedRPE extends the original 3D relative offsets $\left[\Delta x_{ij},\Delta y_{ij},\Delta z_{ij}\right]$ with two surface-variation terms: the normal-angle difference $\Delta \theta_{ij}$ and the curvature difference $\Delta \kappa_{ij}$. As a result, each neighboring pair is represented by a 5D geometry-aware descriptor $\left[\Delta x_{ij},\Delta y_{ij},\Delta z_{ij},\Delta \theta_{ij},\Delta \kappa_{ij}\right]$, which is used to produce an attention bias. The normal-angle difference $\Delta \theta_{ij}$ describes the local orientation change between neighboring points, which is useful near object boundaries and thin structures where adjacent points may be spatially close but belong to different surfaces. The curvature-related difference $\Delta \kappa_{ij}$ reflects local surface variation and helps distinguish planar regions from more irregular geometric structures. Together with the 3D relative offsets, these two terms provide additional surface-variation cues for generating geometry-aware attention bias.

The geometric attributes used by EnhancedRPE are obtained from the preprocessed point-cloud attributes rather than estimated online inside each attention layer. Specifically, the surface normal of point *i* is denoted as ${𝐧}_{i}$. For S3DIS, normals are generated from the raw mesh during preprocessing, while ScanNet provides preprocessed normal attributes in the point-cloud data. When curvature-related cues are enabled, they are computed during preprocessing from local *k*-nearest-neighbor neighborhoods. Given point *i*, let ${𝐩}_{i}\in {\mathbb{R}}^{3}$ denote its 3D coordinate, and let 𝒩(i) denote its local neighborhood, where ${𝐩}_{j}$ denotes the coordinate of a neighboring point j∈𝒩(i). The local centroid and covariance matrix are computed as


$$\begin{array}{c} {\bar{𝐩}}_{i}=\frac{1}{\left|𝒩(i)\right|}\sum_{j\in 𝒩(i)}{𝐩}_{j}, \end{array}$$ (4)

$$\begin{array}{c} \Sigma_{i}=\frac{1}{\left|𝒩(i)\right|}\sum_{j\in 𝒩(i)}({𝐩}_{j}-{\bar{𝐩}}_{i})({𝐩}_{j}-{\bar{𝐩}}_{i}{)}^{⊤}. \end{array}$$ (5)

The eigen-decomposition of $\Sigma_{i}$ is then written as


$$\begin{array}{c} \Sigma_{i}={𝐔}_{i}\Lambda_{i}{𝐔}_{i}^{⊤}, \end{array}$$ (6)

$$\begin{array}{c} \Lambda_{i}=\mathrm{diag}\left(\lambda_{i}^{\left(1\right)},\lambda_{i}^{\left(2\right)},\lambda_{i}^{\left(3\right)}\right), \end{array}$$ (7)

where $\lambda_{i}^{\left(1\right)}\geq \lambda_{i}^{\left(2\right)}\geq \lambda_{i}^{\left(3\right)}\geq 0$. A scalar curvature cue can be computed as


$$\begin{array}{c} \kappa_{i}=\frac{\lambda_{i}^{\left(3\right)}}{\lambda_{i}^{\left(1\right)}+\lambda_{i}^{\left(2\right)}+\lambda_{i}^{\left(3\right)}+\epsilon }, \end{array}$$ (8)

where ϵ is a small constant for numerical stability.

Given a point *i* and its neighboring point j∈𝒩(i), the relative 3D offset is computed as


$$\begin{array}{c} \Delta {𝐩}_{ij}={𝐩}_{j}-{𝐩}_{i}=[\Delta x_{ij},\Delta y_{ij},\Delta z_{ij}{]}^{⊤}. \end{array}$$ (9)

Subsequently, the stored normal vectors ${𝐧}_{i},{𝐧}_{j}$ and curvature values $\kappa_{i},\kappa_{j}$ are obtained for these two points. The pairwise normal-angle and curvature-related differences are computed as


$$\begin{array}{cc} \Delta \theta_{ij} & =\arccos\;\left(\mathrm{clip}\left({𝐧}_{i}^{⊤}{𝐧}_{j},-1,1\right)\right), \end{array}$$ (10)

$$\begin{array}{cc} \Delta \kappa_{ij} & =|\kappa_{j}-\kappa_{i}|, \end{array}$$ (11)

where clip(·) clamps the inner product to [−1,1] for numerical stability. Then, the geometry-aware descriptor in the EnhancedRPE module for a pair of points (*i*,*j*) is constructed as ${𝐫}_{ij}$:


$$\begin{array}{c} {𝐫}_{ij}=[\Delta x_{ij},\Delta y_{ij},\Delta z_{ij},\Delta \theta_{ij},\Delta \kappa_{ij}{]}^{⊤}\in {\mathbb{R}}^{5}. \end{array}$$ (12)

Finally, a lightweight two-layer MLP is applied to ${𝐫}_{ij}$ to produce the pair-wise geometry-aware bias term *R*_ER,*ij*_ as


$$\begin{array}{c} R_{ER,ij}={𝐰}_{2}^{⊤}\sigma ({𝐖}_{1}{𝐫}_{ij}+{𝐛}_{1})+b_{2}, \end{array}$$ (13)

where **W**_1_, **b**_1_, **w**_2_, and *b*_2_ are learnable parameters and σ(·) denotes a non-linear activation. For notational simplicity, the attention-head index is omitted. This bias allows the model to exploit positional offsets, normal-angle differences, and curvature-related contrasts, thereby producing geometry-aware attention weights.

### Conditional multimodal gating module

The Conditional Multimodal Gating (CMG) module introduces a learned channel-wise gating operation before the standard PTv3 input embedding. Given the available input feature representation **F**, CMG first computes global average-pooled and max-pooled channel descriptors. The two descriptors are passed through a shared MLP and combined through a sigmoid function to generate adaptive channel weights:


$$\begin{array}{c} 𝐰=\sigma \;\left(\mathrm{MLP}\;\left(\mathrm{AvgPool}(𝐅)\right)+\mathrm{MLP}\;\left(\mathrm{MaxPool}(𝐅)\right)\right). \end{array}$$ (14)

The input representation is then recalibrated as


$$\begin{array}{c} {𝐅}_{CMG}=𝐅⊙𝐰, \end{array}$$ (15)

where ⊙ denotes channel-wise multiplication.

In GAMPoint, XYZ coordinates provide the basic geometric representation of the point cloud, while auxiliary attributes such as RGB colors and surface normals provide complementary appearance and surface-orientation cues. For RGB-D reconstructed datasets such as ScanNet, the XYZ coordinates already contain depth-derived geometric information, while raw depth is not included as an additional per-point input channel.

When additional aligned feature channels are available, they can be incorporated into the input representation before the same gating operation, allowing the enlarged feature representation to be jointly recalibrated.

Unless otherwise specified, the default setting in the main experiments uses XYZ + RGB+Normal as input, while the input-attribute ablation additionally evaluates XYZ and RGB + XYZ settings. Here, XYZ + RGB+Normal denotes the overall input-attribute setting used in the experiments. Curvature-related cues, when used by EnhancedRPE, are prepared separately during preprocessing and used for attention-bias construction rather than concatenated into the CMG input representation.

After channel-wise recalibration, the resulting features are voxelized and processed by the standard PTv3 input embedding, including a submanifold sparse convolution with kernel size 5×5×5 (SubMConv3d), followed by normalization and a GELU activation. This produces the initial *C*_0_-dimensional feature representation before the serialized encoder, where *C*_0_ = 32.

## Experiments

### Datasets

GAMPoint was evaluated on two publicly available indoor 3D semantic segmentation datasets, ScanNet [57] and S3DIS [58]. ScanNet is an RGB-D video-based indoor scene dataset that provides reconstructed 3D scenes with 20 semantic categories for semantic segmentation. Following the commonly used ScanNet semantic segmentation protocol, we used 1,201 scenes for training and 312 scenes for validation. S3DIS is a room-scale indoor scene dataset collected from six areas across three buildings and contains 271 room-scale scans with 13 semantic categories. Following the standard Area-5 protocol, Areas 1–4 and 6 were used for training, and Area 5 was used for evaluation. Since the number of points varies across scenes after voxelization and crop-based sampling, we report the voxel size and crop-based point limits used in the training pipeline rather than a single fixed point count. Table 2 summarizes the dataset scale, semantic classes, train/evaluation splits, voxel size, and crop-based point limits used in the experiments. All compared methods were evaluated according to the corresponding official or commonly adopted split protocols reported for each dataset.

**Table 2:** Dataset, training, augmentation, and TTA settings on ScanNet and S3DIS.

Group	Item	ScanNet	S3DIS
Dataset and point processing	Dataset type	Indoor RGB-D reconstructed 3D scene dataset	Indoor room-scale 3D scene dataset
	Dataset scale	1,201 training scenes / 312 validation scenes	271 room-scale scans
	Classes	20 semantic categories	13 semantic categories
	Split protocol	Official train / validation split	Areas 1–4 and 6 for training; Area 5 for evaluation
	Evaluation set	Validation set	Area 5
	Voxel size	0.02 m	
	Crop / point limit	SphereCrop; max 102,400 points	SphereCrop ×2; sampling rate = 0.6; max 204,800 points
Training	Hardware	4× RTX 3090 GPUs, 24 GB each	
	Epochs	800	3000
	Batch-size setting	12	
	Workers / AMP	24 / True	
	Optimizer	AdamW, with $\left(\beta_{1},\beta_{2})=(0.9,0.999\right)$	
	Base learning rate	0.006	
	Weight decay	0.05	
	LR schedule	OneCycleLR with cosine annealing	
	Parameter-group max LR	[0.006, 0.0006]	
	Parameter-group setting	head: 0.006 for newly added layers; block: 0.0006 for backbone blocks	
	Warm-up proportion	0.05	
	Initial / final LR scaling factor	10.0 / 1000.0	
	Loss function	CE + Lovász-Softmax	
	Loss weights	1 / 1	
	Ignore index	−1	
Augmentation and TTA	Center shift	Yes, with apply_z; the mean coordinate was subtracted to re-center each scene	
	Point dropout	Applied to 20% of mini-batches, with drop probability of 0.2	
	Random rotation	*p* = 0.5; z∈[−π,π]; x/y∈[−π/64,π/64]	
	Random scaling	Isotropic scale factor sampled from [0.90,1.10]	
	Random flip	*p* = 0.5 along horizontal axes	
	Gaussian noise	Mean = 0; standard deviation = 0.005	
	Color transforms	AutoContrast *p* = 0.2; Translation *p* = 0.95 with ratio = 0.05; Jitter *p* = 0.95 with standard deviation = 0.05	
	Elastic distortion	Yes; coarse [0.2,0.4] and fine [0.8,1.6]	No
	TTA	${\text{rot}}_{z}$: $\left\{0,\frac{\pi }{2},\pi ,\frac{3\pi }{2}\right\}$; scale: {0.95,1.05}; flip-only setting; 13 views in total	scale: {0.90,0.95,1.00,1.05,1.10}; horizontal flip; 10 views in total; predictions averaged
**Note.**		Shared settings are merged across the ScanNet and S3DIS columns. Crop-based point limits were used during training. Although ScanNet is an RGB-D dataset, raw depth was not used as an additional per-point input channel in the reported experiments.	

### Implementation details

Our experiments were implemented in PyTorch. We employed a combination of point-wise cross-entropy loss ${ℒ}_{CE}$ and Lovász-Softmax loss ${ℒ}_{Lovasz}$ [59] as follows:


$$\begin{array}{c} ℒ={ℒ}_{CE}+{ℒ}_{Lovasz}. \end{array}$$ (16)

To balance these two losses, their loss weights (*w*_CE_ and *w*_Lovasz_) were both set to 1. Points that were unlabeled or belonged to ignored categories were assigned the ignore index of −1 and were excluded from loss computation. The models were trained on four NVIDIA RTX 3090 GPUs with 24 GB memory. The main training hyperparameters and dataset-specific settings are summarized in Table 2.

The voxelization and crop-based point limits followed the settings summarized in Table 2. After preprocessing, each scene was stored as arrays such as coordinates, colors, optional normals, semantic labels, instance labels, and offsets, which were then used to construct the sparse-convolutional backbone efficiently, and room-level crops with overlapping inference were employed during test time to follow the standard protocol [8,14,58].

For geometric preprocessing, we followed a Pointcept/PTv3-compatible preprocessing pipeline with optional geometry parsing. For S3DIS, surface normals were generated from the raw mesh when normal parsing was enabled. The released preprocessing script also provides an optional curvature-parsing procedure, where curvature-related descriptors are computed before training by applying PCA/eigenvalue analysis to fixed k-nearest-neighbor neighborhoods with k∈{16,32}. When this option is enabled, the resulting descriptors are stored with the room-level point clouds before training. Therefore, no additional online geometric preprocessing is performed inside each attention layer. In the reported default experiments, the evaluated CMG input setting was XYZ + RGB+Normal, and raw depth was not used as an additional per-point input channel.

Augmentation strategies were applied to improve training robustness. Specifically, we used center shift, random dropout, random rotation, random scaling, random flipping, Gaussian noise, and RGB color transformations during training. For ScanNet, elastic distortion was additionally applied. We also used dataset-specific test-time augmentation: S3DIS used scaling and flipping, while ScanNet used rotation, scaling, and flip-based augmentation. The full data augmentation and test-time augmentation configurations are listed in Table 2.

### Evaluation metrics

Here, *C*_cls_ denotes the number of semantic classes. We adopted mean Intersection over Union (mIoU) and mean Accuracy (mAcc) as evaluation metrics. IoU is widely used to evaluate segmentation quality by measuring region-level overlap and the balance between false positives and false negatives across categories. Thus, the IoU for the class $c\in \left\{1,\cdots ,C_{cls}\right\}$, is calculated as


$$\begin{array}{c} IoU_{c}=\frac{\left|Y_{c}\cap G_{c}\right|}{\left|Y_{c}\cup G_{c}\right|}, \end{array}$$ (17)

The *mIoU* is calculated by averaging the IoU scores of all classes as


$$\begin{array}{c} mIoU=\frac{1}{C_{cls}}\sum_{c=1}^{C_{cls}}IoU_{c}, \end{array}$$ (18)

Accuracy (Acc) measures the ratio of correct classification results for each category. Thus, accuracy is calculated for each class $c\in \left\{1,\cdots ,C_{cls}\right\}$ as


$$\begin{array}{c} Acc_{c}=\frac{TP_{c}}{TP_{c}+FN_{c}}, \end{array}$$ (19)

$FN_{c}$ denotes the number of points from class $c\in \left\{1,\cdots ,C_{cls}\right\}$ that are misclassified as other classes, and $TP_{c}$ denotes the number of correctly classified points of class *c*. The mAcc is calculated by averaging accuracy scores of all classes as


$$\begin{array}{c} mAcc=\frac{1}{C_{cls}}\sum_{c=1}^{C_{cls}}Acc_{c}, \end{array}$$ (20)

The architectural complexity was evaluated using the number of parameters (Params). To provide a more complete efficiency characterization, we further report the observed GPU memory usage and logged evaluation-time statistics under the S3DIS RGB + XYZ+Normal setting. Unless stated, the Params reported of SOTA methods in our experiments were referred from the original paper or the official repository and are used as the canonical baseline.

### Experimental results

To evaluate the comparative performance of GAMPoint, we compared it with representative state-of-the-art (SOTA) methods on ScanNet and S3DIS. These methods employed various architectures.

- Point-based MLP / graph-based methods: PointNet++ [16], PointCNN [41], HPEIN [60], PointWeb [61], and SegGCN [62] model unordered point sets by stacking shared MLPs or graph convolutions over local neighborhoods.
- Voxel- / kernel-based convolutional methods: KPConv [17], SparseConvNet [10], and MinkUNet [2] rely on 3D convolutions (dense or sparse) defined on voxel grids or kernel points to capture local geometric structures.
- Multi-view RGB-D fusion methods: 3dmv [22] aggregates 2D CNN features from multiple RGB-D views into a 3D volumetric representation to jointly exploit image appearance and 3D geometry.
- Transformer-based 3D methods: Swin3D [48] and the Point Transformer family PTv1 [12], PTv2 [13], and PTv3 [14] employ self-attention mechanisms to model long-range dependencies in 3D scenes.

On the S3DIS dataset, GAMPoint achieves 72.1% mIoU and 77.8% mAcc, as shown in Table 3. Compared with PTv3 under a comparable parameter budget, GAMPoint obtains a marginal mIoU gain of 0.1 points and a larger mAcc gain of 1.0 points. These results indicate that the proposed geometry-aware design remains competitive with strong point-transformer baselines, while the overall mIoU improvement over PTv3 should be interpreted as modest. Compared with CNN-based methods such as MinkUNet and KPConv, GAMPoint obtains higher mIoU values by 6.7 and 5.0 points, respectively. It also achieves higher mIoU and mAcc than earlier point-based baselines such as PointNet and PointCNN, as shown in Table 3. These comparisons indicate that GAMPoint remains competitive among representative indoor point-cloud segmentation methods, while the improvement over the strongest PTv3 baseline should still be interpreted as modest.

**Table 3:** Performance comparison among GAMPoint and other SOTA methods on the S3DIS dataset. The performance was evaluated using mIoU (%) and mAcc (%), where σ denotes the standard deviation of class-wise IoU across categories.

Method	Model	Input	mIoU±σ	mAcc	Params
HPEIN [60]	CNN	Point	61.9±27.66	68.3	–
PointWeb [61]	CNN	Point	60.3±27.69	66.6	5.8M
PointCNN [41]	CNN	Point	57.3±29.17	63.9	4.4M
MinkUnet [2]	CNN	Voxel	65.4±26.58	71.7	37.9M
KPConv [17]	CNN	Point	67.1±26.67	72.8	14.9M
SegGCN [62]	CNN	Point	63.6±27.19	70.4	3.0M
PTv1 [12]	ViT	Point	70.4±25.47	76.5	7.8M
PTv2 [13]	ViT	Point	71.6±27.28	77.9	12.8M
PTv3 [14]	ViT	Voxel	72.0±27.36	76.8	46.2M
GAMPoint (ours)	ViT	Voxel	72.1±25.13	77.8	46.2M

On the ScanNet dataset, GAMPoint achieves 77.1% mIoU, as shown in Table 4. Compared with PTv3 under the same parameter budget, GAMPoint improves the mIoU by 0.5 points. This result suggests that incorporating geometry-aware relative positional encoding provides a small positive gain over the PTv3 reference, while maintaining a comparable model size. Additionally, GAMPoint obtains a higher mIoU than the voxel-based transformer Swin3D while using fewer parameters, and achieves mIoU improvements of 4.6 and 3.5 points over SparseConvNet and MinkUNet, respectively. Compared with the previous transformer generation PTv2, GAMPoint also delivered higher mIoU, and it maintained a large margin over early point-based and point-image fusion methods such as PointNet and 3DMV. Overall, the results on ScanNet showed that GAMPoint provided a better trade-off between accuracy and model complexity than both CNN-based and ViT-based competitors.

**Table 4:** Performance comparison among GAMPoint and other SOTA methods on the ScanNet dataset. The performance was evaluated using mIoU (%), where σ denotes the standard deviation of class-wise IoU across categories.

Method	Model	Input	mIoU±σ	Params
PointNet++ [16]	MLP	Point	55.7±18.25	3.5M
PointCNN [41]	CNN	Point	49.8±19.66	20.0M
3DMV [22]	CNN	Multi-view	48.4±15.30	–
KPConv [17]	CNN	Point	68.4±17.28	14.9M
SparseConvNet [10]	CNN	Voxel	72.5±15.12	30.1M
Swin3D [48]	ViT	Voxel	76.4±–	23.6M
MinkUnet [2]	CNN	Voxel	73.6±15.29	37.9M
PTv2 [13]	ViT	Point	75.2±–	12.8M
PTv3 [14]	ViT	Voxel	76.6±12.08	46.2M
GAMPoint (ours)	ViT	Voxel	77.1±12.97	46.2M

Qualitative analysis is provided through representative segmentation results on S3DIS and ScanNet, as shown in Figs 3 and 4. The predictions generally preserve the main scene structure and several fine-grained semantic boundaries, such as wall–floor interfaces and curved surfaces. The visual examples suggest that the geometry-aware bias can help produce more coherent predictions for categories with distinctive geometric patterns, such as large planar surfaces and curved objects. However, these qualitative results should be interpreted together with the class-wise analyses, because the improvements are category-dependent and not uniformly observed across all semantic classes.

**Fig 3:**
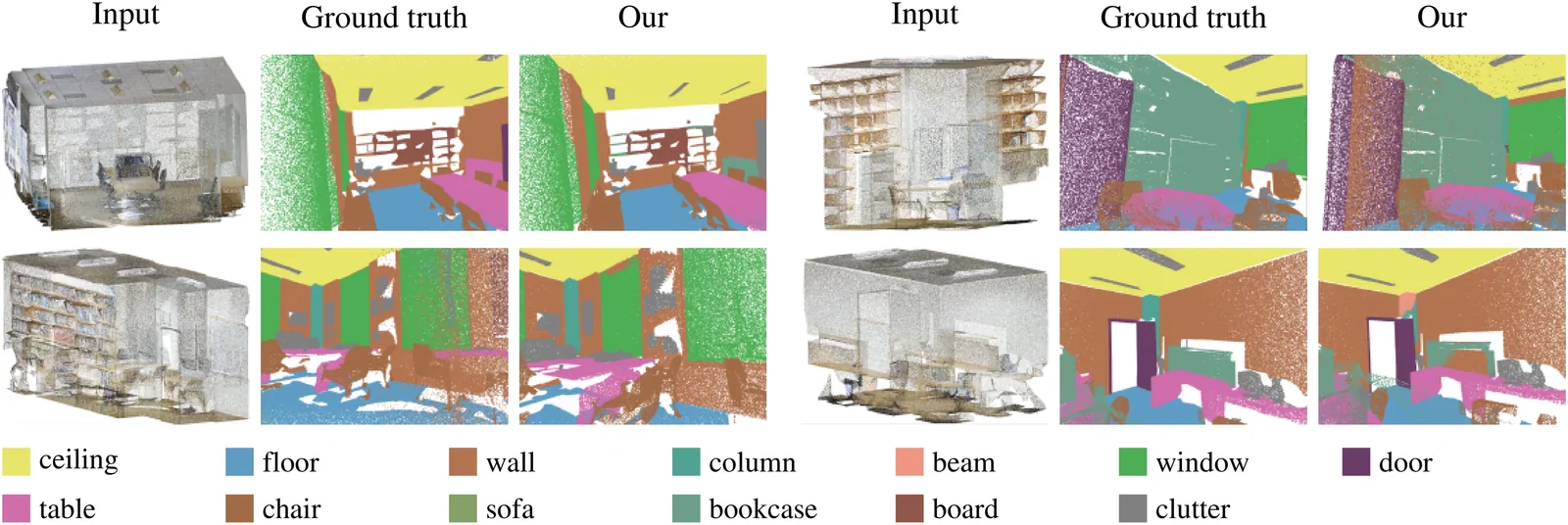
Visualization of semantic segmentation results on S3DIS. Input point clouds, ground truth, and our predictions are shown for representative scenes.

**Fig 4:**
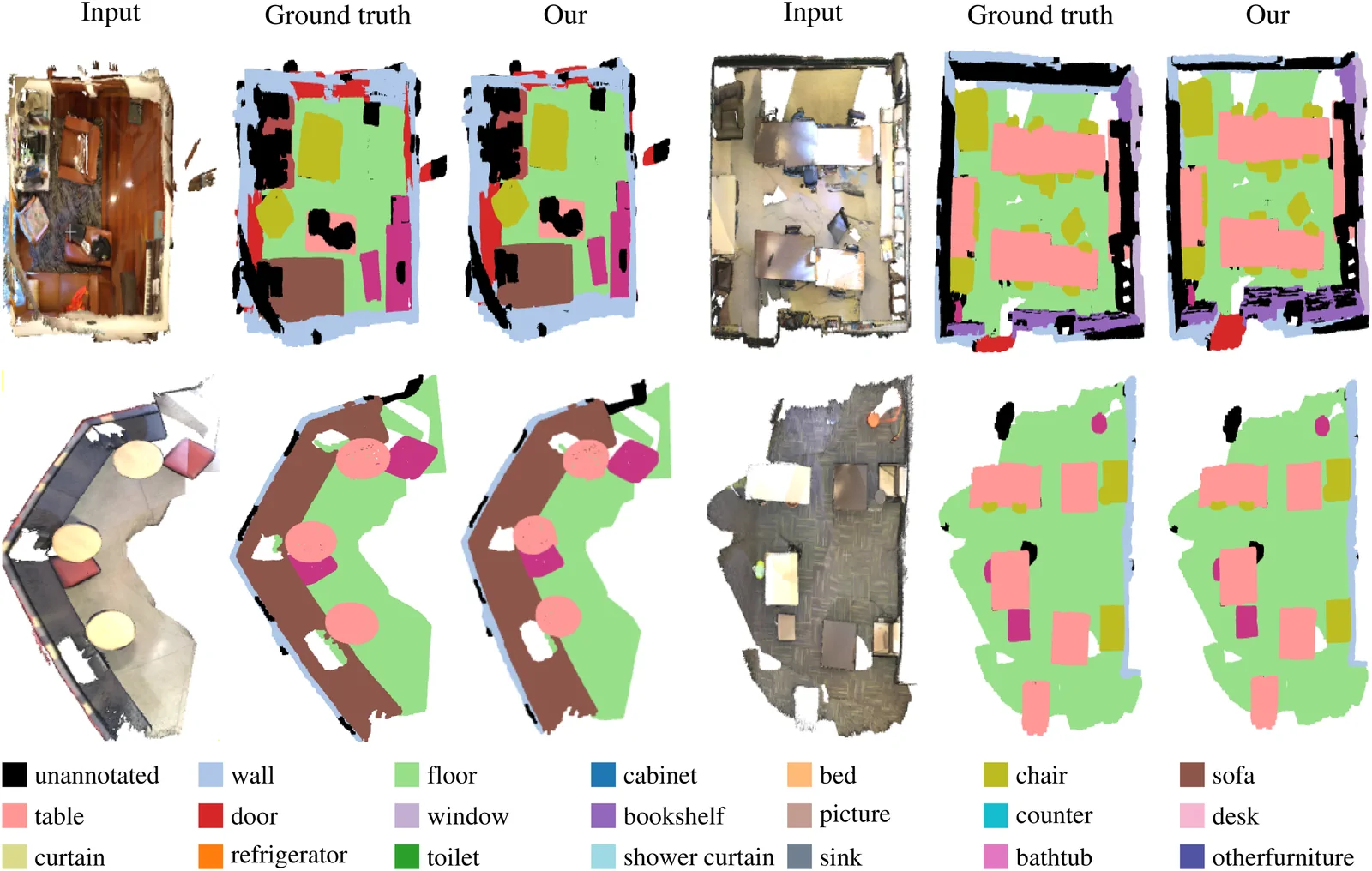
Visualization of semantic segmentation results on ScanNet. Input point clouds, ground truth, and our predictions are shown for representative scenes.

The tested GAMPoint model contains 46.2M parameters. On four RTX 3090 GPUs with 24 GB memory each, the observed training GPU memory usage was approximately 15–21 GiB per GPU. According to the test logs, the average test batch time was 0.057 s for BaseOpt and 0.068 s for GAMPoint, with total evaluation times of approximately 8.0 min and 10.2 min, respectively. These results indicate that GAMPoint introduces a moderate additional evaluation-time cost while remaining compatible with the reported PTv3-based training pipeline. The efficiency and reproducibility information is summarized in Table 5.

**Table 5:** Efficiency and reproducibility summary under the reported S3DIS RGB + XYZ+Normal setting.

Item	BaseOpt	GAMPoint
Input setting	RGB + XYZ+Normal	
Hardware	4 × RTX 3090 GPUs, 24 GB each	
Parameters	46.2M	
Observed training memory	approximately 15–21 GiB/GPU	
Average test batch time	0.057 s	0.068 s
Total evaluation time	approximately 8.0 min	approximately 10.2 min

Table 6 reports the per-class IoU and accuracy comparison between BaseOpt and GAMPoint on S3DIS Area-5. GAMPoint changes the overall mIoU from 72.00% to 72.12% and the overall mAcc from 76.85% to 77.80%. The largest positive change is observed for column, while decreases are observed for categories such as door and board. These results show that the improvement is category-dependent rather than uniform.

**Table 6:** Per-class IoU and accuracy comparison on S3DIS Area-5 between BaseOpt and GAMPoint. Per-class IoU (%) and accuracy (%) are reported; ΔIoU and ΔAcc denote GAMPoint-BaseOpt.

Class	BaseOpt		GAMPoint		Difference	
	IoU	Acc	IoU	Acc	ΔIoU	ΔAcc
ceiling	92.90	94.81	93.11	95.10	+0.21	+0.29
floor	98.32	99.15	98.50	99.26	+0.18	+0.11
wall	85.24	97.82	85.85	97.84	+0.61	+0.02
beam	0	0	0	0	–	–
column	26.53	27.16	46.27	49.40	+19.74	+22.24
window	65.48	67.71	62.89	65.13	−2.59	−2.58
door	76.44	85.20	70.04	81.40	−6.40	−3.80
table	83.97	91.32	83.68	90.07	−0.29	−1.25
chair	92.92	97.09	92.87	96.55	−0.05	−0.54
sofa	81.99	84.49	81.60	84.44	−0.39	−0.05
bookcase	80.63	88.64	79.32	85.22	−1.31	−3.42
board	88.70	91.26	80.91	91.54	−7.79	+0.28
clutter	62.95	74.38	62.47	75.45	−0.48	+1.07
**Overall**	**72.00**	**76.85**	**72.12**	**77.80**	**+0.12**	**+0.95**

Table 7 reports the per-class IoU comparison between BaseOpt and GAMPoint on ScanNet. GAMPoint improves the overall mIoU from 76.60% to 77.10%. The largest gain is observed for chair, followed by counter, curtain, cabinet, and bed, whereas categories such as refrigerator, desk, and picture decrease. Therefore, the per-class results provide a more balanced view of the overall improvement.

**Table 7:** Per-class IoU (%) on ScanNet between BaseOpt and GAMPoint under the same training and evaluation protocol used for the main benchmark.

Class	BaseOpt IoU	GAMPoint IoU	Difference
wall	87.37	87.21	−0.16
floor	96.44	96.27	−0.17
cabinet	71.12	73.80	+2.68
bed	83.53	85.86	+2.33
chair	82.21	92.01	+9.80
sofa	84.11	86.15	+2.04
table	80.35	79.45	−0.90
door	73.29	72.29	−1.00
window	68.67	70.11	+1.44
bookshelf	82.18	83.91	+1.73
picture	41.23	38.56	−2.67
counter	65.60	71.43	+5.83
desk	73.77	70.84	−2.93
curtain	74.77	77.40	+2.63
refrigerator	71.29	66.79	−4.50
shower curtain	70.30	71.51	+1.21
toilet	93.47	94.21	+0.74
sink	71.22	69.21	−2.01
bathtub	90.06	90.61	+0.55
otherfurniture	63.19	64.87	+1.68
**Overall**	**76.60**	**77.10**	**+0.50**

#### Repeated-run analysis.

To assess run-to-run variability, we evaluated BaseOpt and GAMPoint using five matched random seeds, with the same seed used for both models within each pair, as summarized in Table 8. On S3DIS, BaseOpt achieved 71.40 ± 0.38% mIoU and 76.52 ± 0.42% mAcc, whereas GAMPoint achieved 72.07 ± 0.36% mIoU and 77.61 ± 0.25% mAcc. The mean paired improvements were +0.67 and +1.09 percentage points for mIoU and mAcc, respectively, and both differences were statistically significant under two-sided paired *t*-tests (*p* < 0.05). On ScanNet, BaseOpt and GAMPoint achieved 76.75 ± 0.17% and 77.12 ± 0.25% mIoU, respectively, corresponding to a mean paired improvement of +0.37 percentage points, which was also statistically significant (*p* < 0.05). All five matched ScanNe*t* comparisons showed a positive mIoU difference in favor of GAMPoint. The single-run values reported in the main benchmark and class-wise tables correspond to the original reference run, whereas the repeated-run results report the mean ± standard deviation across five matched runs. The S3DIS GAMPoint reference run is included among these five runs and achieves 72.12% mIoU, compared with the five-run mean of 72.07 ± 0.36%.

**Table 8:** Repeated-run analysis of BaseOpt and GAMPoint using five matched random seeds. Values are reported as mean ± standard deviation across five matched runs. The same random seed was used for BaseOpt and GAMPoint within each pair, and statistical significance was evaluated using two-sided paired *t*-tests.

Dataset	Metric	Runs	BaseOpt	GAMPoint	Mean paired difference	*p*-value
S3DIS	mIoU	5	71.40 ± 0.38	72.07 ± 0.36	+0.67	< 0.05
S3DIS	mAcc	5	76.52 ± 0.42	77.61 ± 0.25	+1.09	< 0.05
ScanNet	mIoU	5	76.75 ± 0.17	77.12 ± 0.25	+0.37	< 0.05

### Ablation study

#### Overall EnhancedRPE ablation.

To evaluate the overall contribution of EnhancedRPE, we first compare the complete GAMPoint with the corresponding variant in which EnhancedRPE is disabled. On S3DIS, the mIoU increases from 71.90% to 72.12%, while on ScanNet it increases from 76.83% to 77.10%. The class-wise results in Tables 9 and 10 further show that the influence of EnhancedRPE is category-dependent. Compared with the variant without EnhancedRPE, GAMPoint improves several structure-sensitive categories, such as column and board on S3DIS and cabinet and counter on ScanNet, whereas several categories show decreases. These results indicate that EnhancedRPE provides useful local geometric cues for some categories, but it does not uniformly improve all semantic classes. The decreased categories can be regarded as representative weak cases of the current design. They suggest that categories dominated by appearance patterns, object-level context, or global scene layout may not benefit sufficiently from local surface-variation cues alone.

**Table 9:** Ablation study of the proposed EnhancedRPE module on S3DIS. Per-class IoU (%) and overall mIoU (%) are reported; Difference denotes GAMPoint minus the variant without EnhancedRPE.

Class	GAMPoint w/o EnhancedRPE	GAMPoint	Difference
ceiling	92.27	93.11	+0.84
floor	98.21	98.50	+0.29
wall	86.07	85.85	−0.22
column	38.15	46.27	+8.12
window	63.38	62.89	−0.49
door	76.13	70.04	−6.09
table	85.47	83.68	−1.79
chair	93.26	92.87	−0.39
sofa	85.19	81.60	−3.59
bookcase	79.95	79.32	−0.63
board	76.10	80.91	+4.81
clutter	60.51	62.47	+1.96
Overall	71.90	72.12	+0.22

**Table 10:** Ablation study of the proposed EnhancedRPE module on ScanNet. Per-class IoU (%) and overall mIoU (%) are reported; Difference denotes GAMPoint minus the variant without EnhancedRPE.

Class	GAMPoint w/o EnhancedRPE	GAMPoint	Difference
wall	87.37	87.21	−0.16
floor	96.36	96.27	−0.09
cabinet	71.94	73.80	+1.86
bed	84.33	85.86	+1.53
chair	92.36	92.01	−0.35
sofa	83.71	86.15	+2.44
table	78.52	79.45	+0.93
door	74.19	72.29	−1.90
window	70.58	70.11	−0.47
bookshelf	81.95	83.91	+1.96
picture	36.20	38.56	+2.36
counter	67.61	71.43	+3.82
desk	71.41	70.84	−0.57
curtain	78.87	77.40	−1.47
refrigerator	71.16	66.79	−4.37
shower curtain	72.89	71.51	−1.38
toilet	94.14	94.21	+0.07
sink	69.64	69.21	−0.43
bathtub	91.96	90.61	−1.35
otherfurniture	61.44	64.87	+3.43
Overall	76.83	77.10	+0.27

#### Component-wise EnhancedRPE ablation.

To further isolate the contributions of the individual geometric terms, we conducted a component-wise ablation on S3DIS with CMG disabled in all variants and with the input attributes and training and evaluation settings kept unchanged, as summarized in Table 11. The original PTv3 coordinate-based RPE using the 3D relative offsets serves as the common baseline. The coordinate offsets are retained in all variants, while the normal-angle difference and curvature difference are introduced individually and then jointly. The coordinate-based RPE achieves 72.00% mIoU and 76.85% mAcc. Adding the normal-angle term increases the results to 72.08% mIoU and 77.49% mAcc, while adding the curvature-difference term yields 72.05% mIoU and 77.73% mAcc. The full EnhancedRPE, combining the coordinate offsets with both surface-variation terms, achieves the highest mIoU of 72.10%. These results indicate that both proposed surface cues provide modest incremental benefits beyond the coordinate-based RPE, while their combination gives the best overall mIoU.

**Table 11:** Component-wise ablation of the geometric terms in EnhancedRPE on S3DIS. All variants in this component-wise ablation are evaluated with CMG disabled, while the input attributes and training and evaluation settings are kept unchanged.

Variant	Δxyz	Δθ	Δκ	mIoU (%)	mAcc (%)
Coordinate-based RPE	✓	–	–	72.00	76.85
+ Normal-angle	✓	✓	–	72.08	77.49
+ Curvature-difference	✓	–	✓	72.05	77.73
Full EnhancedRPE	✓	✓	✓	**72.10**	**77.80**

#### Serialized attention-window ablation.

We further examined the influence of the serialized attention-window size on S3DIS under the RGB + XYZ+Normal setting. In addition to the default setting *w* = 1024 used in all main experiments, two alternative window sizes, *w* = 512 and *w* = 2048, were evaluated over three runs. The average mIoU/mAcc/allAcc values were 71.86%/77.10%/91.24% for *w* = 512 and 72.12%/77.37%/91.22% for *w* = 2048, respectively. These results indicate that the model is not highly sensitive to the tested window-size range. A smaller window slightly reduced mIoU, whereas a larger window did not provide a clear overall advantage across all metrics and increases the attention-window scale. Therefore, *w* = 1024 was retained as the default setting for all main comparisons, following the PTv3-based baseline configuration and providing a practical balance between accuracy and computational cost.

#### CMG and input-attribute ablation.

To directly evaluate the contribution of CMG, we additionally include a CMG-only variant, implemented as GAMPoint without EnhancedRPE, and compare it with BaseOpt under the same three input settings. BaseOpt represents the standard PTv3 input pathway, including ordinary channel-wise concatenation when multiple attributes are available, whereas the CMG-only variant additionally introduces learned channel-wise recalibration before the PTv3 embedding. The corresponding results are summarized in Table 12.

**Table 12:** Controlled ablation of CMG under different input-attribute settings on S3DIS. GAMPoint w/o EnhancedRPE denotes the CMG-only variant.

Method	Input	mIoU (%)	mAcc (%)
BaseOpt	XYZ	68.56	74.27
GAMPoint w/o EnhancedRPE	XYZ	68.58	74.59
GAMPoint	XYZ	69.04	74.19
BaseOpt	RGB + XYZ	70.77	75.41
GAMPoint w/o EnhancedRPE	RGB + XYZ	70.80	76.01
GAMPoint	RGB + XYZ	70.85	75.87
BaseOpt	RGB + XYZ+Normal	72.00	76.85
GAMPoint w/o EnhancedRPE	RGB + XYZ+Normal	71.90	77.97
GAMPoint	RGB + XYZ+Normal	72.12	77.80

Compared with BaseOpt, the CMG-only variant increases mAcc by 0.32, 0.60, and 1.12 percentage points under the XYZ, RGB + XYZ, and RGB + XYZ+Normal settings, respectively. Its corresponding mIoU changes are + 0.02, + 0.03, and −0.10 percentage points. These results show that CMG has a measurable independent effect on the input representation, with consistent mAcc improvements across the evaluated input settings, although its isolated effect on mIoU is modest and input-dependent. We further examine the full RGB + XYZ+Normal setting on ScanNet. BaseOpt achieves 76.60% mIoU, the CMG-only variant achieves 76.83%, and the complete GAMPoint with both CMG and EnhancedRPE achieves 77.10%. Thus, CMG alone provides a 0.23-point mIoU improvement on ScanNet, while the complete CMG+EnhancedRPE configuration provides a 0.50-point improvement over BaseOpt. These results indicate that CMG provides a measurable, dataset- and metric-dependent contribution under the evaluated point-associated attributes. In the current S3DIS and ScanNet pipelines, CMG is evaluated using XYZ, RGB, and surface normals. The implemented input interface can also accommodate additional aligned feature channels before the same learned gating operation, providing a practical basis for extension to richer multimodal settings.

## Discussion

GAMPoint is designed for indoor 3D point-cloud semantic segmentation, which is relevant to applications such as indoor scene understanding, robotic navigation, BIM-oriented reconstruction, and AR/VR scene modelling. In these scenarios, reliable recognition of structural components and object boundaries is important for downstream understanding. The class-wise analyses in Tables 6, 7, 9, and 10 show that the gains are category-dependent rather than uniformly distributed across all semantic classes.

This pattern is consistent with the motivation of EnhancedRPE and the qualitative observations in Figs 3 and 4. Standard coordinate-based relative positional encoding mainly describes spatial offsets between neighboring points, whereas EnhancedRPE further introduces local surface-orientation and surface-variation cues. These cues can help distinguish neighboring points that are spatially close but belong to different surfaces, especially near object boundaries, wall-floor interfaces, curved surfaces, and thin structures. However, categories that depend more strongly on appearance, object-level context, or global layout may benefit less from local surface-variation cues alone. Therefore, GAMPoint should be interpreted as providing a modest overall improvement with category-dependent gains, rather than as uniformly improving every semantic class.

For the multimodal design, CMG is used as an input-side embedding interface for combining point coordinates with available auxiliary point attributes. In the reported experiments, the evaluated attributes are limited to XYZ coordinates, RGB colors, and normals. For ScanNet, the point coordinates are reconstructed from RGB-D scans and therefore already contain depth-derived geometric information, but raw depth values are not used as an additional per-point input channel. Accordingly, CMG is described as a multimodal-ready input interface rather than as an empirically validated missing-modality or raw-depth fusion framework. It should also be noted that the normal vectors and curvature-related cues discussed in this work are geometry-derived attributes computed from the point cloud itself, rather than independent sensor modalities. In the reported default experiments, curvature-related cues were not used as additional CMG input channels; instead, the evaluated CMG input setting was limited to XYZ + RGB+Normal. The role of CMG in the current study is to provide a prepared input-side interface for future extension to richer multimodal data, such as explicit per-point depth, intensity, thermal cues, or aligned image features. These extensions require dedicated multimodal datasets and separate empirical validation. Future work will also investigate missing-modality robustness, outdoor or cross-domain point-cloud datasets, and real-time inference optimization for deployment-oriented 3D scene understanding.

## Conclusion

In this paper, we propose GAMPoint, a transformer-based framework for 3D point cloud semantic segmentation in complex indoor scenes. GAMPoint is built upon Point Transformer V3 by integrating (i) EnhancedRPE to augment relative positional modeling with local surface-variation cues and (ii) a CMG-based input embedding that is multimodal-ready for fusing optional auxiliary channels. The reported experiments use XYZ + RGB+Normal as input, and raw-depth fusion is left for future work. Experiments on ScanNet and S3DIS show that GAMPoint achieves competitive performance and provides modest gains over strong point-transformer baselines. The class-wise and repeated-run analyses further indicate that the proposed design provides category-dependent improvements and consistent gains across matched repeated runs, although the gains are not uniformly distributed across all semantic classes.
